# The Impact of the Coexistence of Frailty Syndrome and Cognitive Impairment on Early and Midterm Complications in Older Patients with Acute Coronary Syndromes

**DOI:** 10.3390/jcm13237408

**Published:** 2024-12-05

**Authors:** Radosław Wontor, Magdalena Lisiak, Maria Łoboz-Rudnicka, Bogusława Ołpińska, Rafał Wyderka, Krzysztof Dudek, Krystyna Łoboz-Grudzień, Joanna Jaroch

**Affiliations:** 1 Department of Cardiology, Marciniak Lower Silesian Specialist Hospital—Emergency Medicine Center, 54-049 Wroclaw, Poland; rmwontor@gmail.com (R.W.); marialoboz@o2.pl (M.Ł.-R.); olpinskab@gmail.com (B.O.); rafal.wyderka@pwr.edu.pl (R.W.); kloboz@wp.pl (K.Ł.-G.); joanna.jaroch@pwr.edu.pl (J.J.); 2 Department of Nursing, Faculty of Nursing and Midwifery, Wroclaw Medical University, 51-618 Wroclaw, Poland; 3 Institute of Heart Diseases, University Hospital, 50-556 Wroclaw, Poland; 4 Faculty of Medicine, Wroclaw University of Science and Technology, 50-370 Wroclaw, Poland; 5 Faculty of Mechanical Engineering, Wroclaw University of Science and Technology, 50-370 Wroclaw, Poland; krzysztof.dudek@pwr.edu.pl

**Keywords:** frailty syndrome, cognitive impairment, acute coronary syndrome

## Abstract

**Background/Objectives:** The ageing population has heightened interest in the prognostic role of geriatric conditions, notably frailty syndrome (FS) and cognitive impairment (CI). Evidence indicates a significant link between cardiovascular disease, FS, and CI. However, limited research has explored the impact of impaired functional and cognitive performance on outcomes in acute coronary syndrome (ACS) patients. This study aimed to evaluate the effect of coexisting FS and CI (FSxCI) on early and 6-month complications in older adults with ACS. **Methods**: This study included 196 ACS patients (119 men) aged 65 and over (mean = 74.7 years), with 90.8% undergoing invasive treatment (PCI in 81.6%, CABG in 9.2%). FS and CI were assessed on the third hospital day using the Tilburg Frailty Indicator (TFI) and Mini Mental State Examination (MMSE). Early (in-hospital) complications included major bleeding, ventricular arrhythmia (VT), conduction disturbances, cardiac arrest, stent thrombosis, acute heart failure (Killip–Kimball class III/IV), stroke, prolonged stay, and in-hospital death. Six-month follow-up recorded major adverse cardiovascular and cerebrovascular events (MACCEs). **Results**: Patients with FSxCI (n = 107, 54.6%) were older and had higher hypertension prevalence and lower nicotine dependence. FSxCI patients faced over twice the risk of prolonged hospital stays (OR 2.39; *p* = 0.01) and nearly three times the risk of early complications (OR 2.73; *p* < 0.001). At 6 months, FSxCI tripled the risk of MACCEs (OR 2.8; *p* = 0.007). Kaplan–Meier analysis confirmed a worse 6-month prognosis for FSxCI patients. **Conclusions**: Elderly patients with ACS and concomitant FSxCI had significantly higher rates of early (in-hospital) and 6-month complications. FSxCI was associated with a worse 6-month prognosis. This highlights its significance for clinical decision-making, as identifying FSxCI in ACS patients can help prioritize high-risk individuals for tailored interventions, optimize resource allocation, and improve outcomes.

## 1. Introduction

The number of people aged 65 or over with acute coronary syndrome (ACS) is growing due to demographic trends and the epidemiology of coronary heart disease. Appropriate risk stratification is an increasing challenge for cardiologists. Chronological age has been recognized as a strong risk factor for complications and poor prognosis in patients with ACS and is used in commonly recommended risk scales. However, chronological age does not always reflect biological age, which seems to be a more accurate measure of the patient’s condition. Recently, there has been increasing interest in geriatric conditions, including frailty syndrome (FS) and cognitive impairment (CI) and their prognostic role in patients with cardiovascular disease. FS is the state of decreased reserve and increased vulnerability to stressors due to age-associated impairment of general body function [1,2]. Cognitive impairment is a complex entity that might have various manifestations and intensity of intellectual decline, with dementia as its most advanced stage. For the simultaneous presence of FS and mild CI, a term “cognitive frailty” was established by The International Academy on Nutrition and Aging (IANA) and the International Association of Gerontology and Geriatrics (IAGG) [3].

Studies have demonstrated that FS has a negative impact on prognosis in ACS patients [4,5]. However, the knowledge on the association between CI or the coexistence of FS and CI on prognosis in patients with ACS is still insufficient [6,7].

There is a growing body of evidence that cardiovascular disease (CVD), FS, and CI are involved in a vicious circle, in which each of these entities promote and accelerate the progression of the other ones. CI, FS, and CVD share common risk factors like diabetes, hypertension, obesity, and smoking and also share pathophysiologic pathways including systemic inflammation, oxidative stress, and neuroendocrine dysregulation. Moreover, CVD, particularly manifesting in brain vessels, constitutes itself a risk factor for CI. The interaction between CI and CVD is complex and bidirectional. On the one hand, CVD—in particular, cerebrovascular disease—leads to CI; on the other hand, CI, through various mechanisms including, e.g., worse compliance with treatment, may accelerate the progress of CVD and worsen prognosis [8].

Findings from recent studies highlight that the interaction between age, comorbidities, and outcomes in older adults with ACS is particularly pronounced in patients aged 90 and above. Percutaneous coronary interventions (PCIs) are commonly performed for urgent indications in this group. It is worth noting that this group experiences high in-hospital mortality, predominantly due to cardiac causes. Post-discharge outcomes are frequently influenced by non-cardiac conditions, and the long-term prognosis remains poor [9]. As life expectancy increases, the prevalence of ACS in older people with CI continues to rise, highlighting the importance of integrating the assessment of FS and CI into treatment plans. A holistic approach is essential to improve prognosis and quality of life in this population.

There are numerous tools utilized in the diagnostic process of FS and CI. The most popular scales used for FS diagnosis include the Fried Frailty Scale, the FRAIL scale, the frailty index, the Tilburg Frailty Indicator (TFI), and the Edmonton Frailty Scale. In our study, the Tilburg Frailty Indicator was used, as it is a multidimensional model, assessing three frailty domains (physical, psychological, and social) based on a simple validated questionnaire that is easy to perform at bedside in an acute setting of ACS hospitalization [10]. For cognitive function assessment, the Mini Mental State Examination (MMSE), Telephone Interview for Cognitive Status, or Montreal Cognitive Assessment Test are frequently used—in our study, we decided to utilize the MMSE because of its simplicity and local adaptation [11].

Because of the growing population of elderly people with ACS, it is of crucial importance to establish the prognostic role of FS, CI, and their coexistence in this group of patients. In our study, we decided to include not only patients with cognitive frailty (FS and mild CI) but also patients with dementia, except for those with severe dementia, who were incapable of completing questionnaires.

## 2. Materials and Methods

### 2.1. Patient Population and Procedure

The study included 196 patients hospitalized in the Cardiology Department of the T. Marciniak Lower Silesian Specialist Hospital [Pol. Dolnośląski Szpital Specjalistyczny im. T. Marciniaka] in Wroclaw between 2013 and 2019 with a main diagnosis of ACS (STEMI n = 98, NSTE-ACS n = 98). The participants included 119 men and 77 women aged between 65 and 97 (mean age = 74.4 years). The inclusion criteria were as follows: age ≥ 65 years, acute coronary syndrome diagnosed based on the ESC criteria, and the provision of informed consent to participate in the study. Invasive management was performed in 90.8% of the patients (81.6% received PCI and 9.2% underwent coronary artery bypass graft (CABG) surgery). Patients were excluded if they had any mental condition preventing them from completing the questionnaires.

The participants were informed about the purpose and procedure of the study and provided written informed consent to complete the TFI and MMSE questionnaires.

The study was approved by the Bioethics Committee of Wrocław Medical University.

We analyzed selected clinical, demographic, and anamnestic data, laboratory and imaging test results, as well as information on the occurrence of early (in-hospital) complications, including the following:

(a) TIMI major bleeding;
(b) Ventricular arrhythmia;
(c) Cardiac conduction disturbances requiring cardiac stimulation;
(d) Cardiac arrest;
(e) Early stent thrombosis;
(f) Acute heart failure (Killip–Kimball class III/IV);
(g) Stroke;
(h) Prolonged hospital stay (>8 days);
(i) In-hospital death.

At 6-month follow-up, we obtained information on the occurrence of late cardiovascular complications, i.e., a composite endpoint—major adverse cardiovascular and cerebrovascular events (MACCEs)—by means of phone contact. The composite endpoint was defined as any of the following:

(a) Recurrent ACS;
(b) Recurrent revascularisation;
(c) Stroke;
(d) Death.

### 2.2. Measurements

The patients were evaluated for FS on day 3 of their hospital stay using a Polish language adaptation of the TFI questionnaire [12]. Patients with a TFI score of >4 were diagnosed with FS. A TFI score of 5–8 was considered to be indicative of mild FS, whereas a TFI score of 9 or above was considered to indicate moderate or severe FS. At the same time, the patients were screened for CI using a Polish language adaptation of the MMSE scale [13]. An MMSE score of less than 27 was considered to indicate CI (a score of 24–26 was considered to indicate CI without dementia and a score of less than 24 was considered to indicate CI with dementia). Patients with concomitant FS and CI were included in the FSxCI group.

### 2.3. Statistical Analysis

The results of clinical tests were analyzed statistically using STATISTICA 13 (TIBCO Software Inc., Palo Alto, CA, USA) and an EXCEL spreadsheet (Microsoft). All quantitative variables were tested for normal distribution using the Kolmogorov–Smirnov test with Lilliefors significance correction or the Shapiro–Wilk test depending on sample size. The significance threshold was set at *p* < 0.05. The assumption of homogeneity of variance was tested using Levene’s test. Quantitative variables are reported in tables as means (*M*), standard deviations (*SD*), medians (*Me*), lower (*Q*1) and upper (*Q*3) quartiles, and extreme (*Min*. and *Max*.) values. Qualitative (nominal and ordinal) variables are reported in contingency tables as numbers (*n*) and population proportions (%). If an association was found between two variables, the odds ratio (OR) and its 95% confidence interval were also calculated. The significance of differences between mean values in two groups for quantitative variables with a normal distribution was tested using the *t*-test. If the assumption of normality of distribution was not met, the Mann–Whitney U test was performed. Cut-off values for continuous variables (e.g., *Age*) determining the FSxCI were determined using ROC (receiver operating characteristic) curve analysis. The results are reported as odds ratios and their 95% confidence intervals. The impact of the independent variables analyzed (e.g., SFxCI) on survival (6-month mortality) was assessed using Kaplan–Meier analysis. The results are presented as survival curves. The results of the statistical tests performed were considered significant if the probability (*p*) value was less than 0.05.

## 3. Results

### 3.1. Characteristics of the Study Group

Frailty syndrome (TFI score > 4) was present in 137 patients (69.9%), of whom 83 (42.3%) had mild FS (TFI score of 5–8) and 54 (27.6%) had moderate or severe FS (TFI score ≥ 9). Cognitive impairment (MMSE score < 27) was diagnosed in 126 patients (64.3%), of whom 51 (26%) had CI without dementia (MMSE score of 24–26), 57 (29.1%) had mild dementia (MMSE score of 19–23), and 18 (9.2%) had moderate dementia (MMSE score of 11–18). Patients with coexisting FS (TFI score > 4) and CI (MMSE score < 27) were included in the FSxCI group (*n* = 107, 54.6%).

Patients with FSxCI were older than patients without FSxCI (77.9 ± 8.1 vs. 70.8 ± 5.8; *p* < 0.001). Compared to patients without FSxCI, patients with FSxCI were less likely to have nicotine dependence (14.0% vs. 29.2%; *p* = 0.013) and more likely to suffer from hypertension (81.3% vs. 61.8%; *p* = 0.004). There were no differences between the two groups in the rate of invasive management, the rate of optimal percutaneous coronary intervention (PCI) results, and pre-hospital delay to PCI (Table 1).

**Figure 1:**
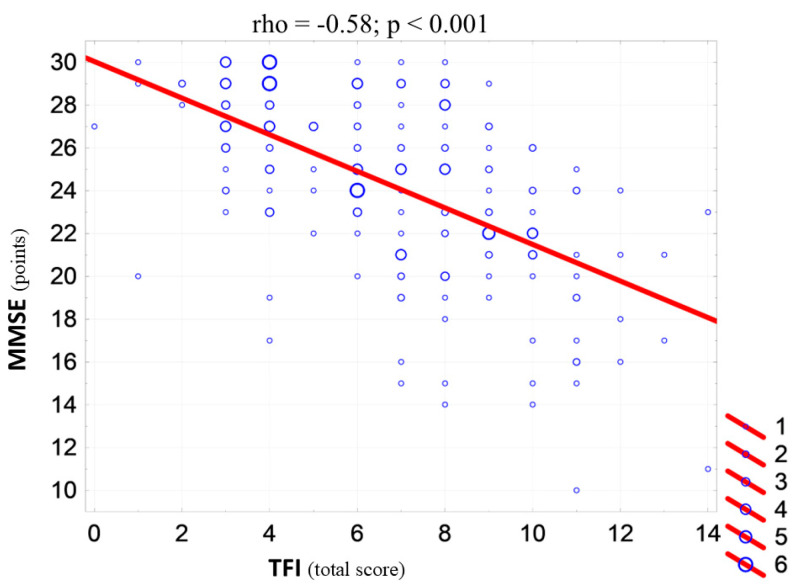
Correlation diagram between the frailty (TFI) and CI (MMSE) scores of the patients studied (n = 196) and Spearman’s rank correlation coefficient (*rho*) value.

In a multivariate analysis, age and hypertension were independent predictors of the FSxCI (Table 2). We found a strong correlation between the severity of FS and the level of CI assessed during the hospitalization. The greater the severity of FS (the higher the TFI score), the greater the severity of CI (the lower the MMSE score). An increase in the TFI score by one point is associated with a reduction in the MMSE score by an average of 0.85 points (Figure 1).

### 3.2. Early Complications

Compared to patients without FSxCI, patients with FSxCI had more than twice the odds of a prolonged hospital stay (OR 2.39 [95% CI: 1.33–4.30]) and almost three times the odds of experiencing early (hospital) complications (OR 2.73 [95% CI: 1.53–4.88]) (Table 3).

### 3.3. Major Adverse Cardiovascular and Cerebrovascular Events (MACCEs) at 6 Months

**Figure 2:**
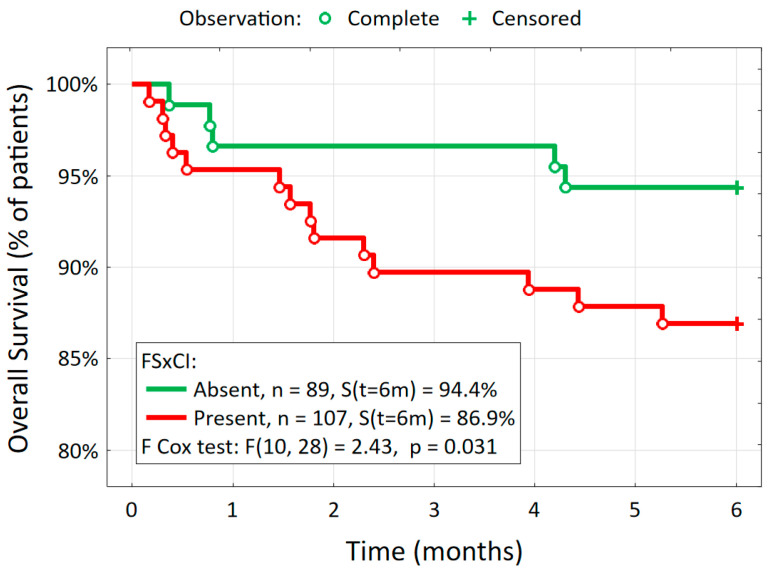
Kaplan–Meier survival curve of patients with and without FSxCI.

Table 3 also shows the analysis of the major adverse cardiovascular and cerebrovascular event rate at 6 months of follow-up according to the presence or absence of FSxCI. The rate of total MACCEs was higher in patients with FSxCI than in those without FSxCI (16.8% vs. 6.7%; *p* = 0.047). FSxCI was associated with an almost threefold increased risk of MACCEs (OR: 2.80 [95% CI: 1.06–7.39]). There was no statistically significant difference between the group with and without FSxCI in the rate of a single adverse event, such as myocardial infarction, repeat revascularization, stroke, or death. Kaplan–Meier curves showed a significantly increased 6-month mortality in patients with FSxCI (*p* = 0.031) (Figure 2).

## 4. Discussion

A growing body of evidence indicates that CI, FS, and CVD are in a strong relationship. Our study shows that in patients >65 years with ACS, the coexistence of FS and CI (FSxCI) is frequent and is associated with an almost threefold increased risk of early complications (mainly prolonged hospital stay) and an almost threefold increased risk of major adverse cardiovascular and cerebrovascular events (MACCEs) at 6 months. Furthermore, FSxCI is associated with a worse 6-month prognosis.

*The prevalence of FS and CI in patients with ACS:* The prevalence of both CI and FS in our study group was high. CI assessed by the MMSE was found in 64.3% of patients and FS assessed by the TFI was found in 69.9% of patients. The reported prevalence of CI and FS varies significantly between the studies. A systematic review by Collard et al., which analyzed 21 studies including a total of 61,500 patients aged 65 and over, found that the reported prevalence rates of FS ranged between 4.0% and 59.1% [14]. A similar divergence was observed for the diagnosis of CI—in a systematic review by Zhao et al., including a total of 6457 patients, the reported prevalence rates of CI ranged between 9 and 85% [15]. Such huge differences in the reported prevalence of CI and FS result from multiple factors, including various studied populations and the use of different frailty and cognitive function assessment tools. Of note, in the study by Kasprzak et al., in which the CI was assessed with the same tool as in our study (MMSE) and which was performed in an ethnically identical, though younger population in comparison to our study (mean age: 59 y vs. 74 y), the prevalence of CI was also significant and stood at 37% [16].

In the present study, we found a strong correlation between the severity of FS and the level of CI. An increase in the TFI score by one point was associated with a reduction in the MMSE score by an average of 0.85 points. A similar association was reported in a study by Sanchis et al., in which CI was found to be influenced by frailty, older age, and female sex [7]. A study by Borges et al. also demonstrated an association between frailty and CI [17]. It has been reported that frail individuals are at higher risk of CI, particularly vascular dementia [18,19,20].

*Early complications:* The literature provides evidence that the association of CI and worse in-hospital outcomes refers not only to cardiovascular hospitalizations but is more universal—a large retrospective analysis of 21,399 incident emergency admissions for various diagnoses (cardiovascular, traumatic, infectious, etc.) of elderly patients revealed that CI is a predictor of prolonged hospital stay (12 days vs. 5 days) and increased in-hospital mortality (12.6% vs. 6.5%) [21]. Our study demonstrates that the coexistence of FS and CI in patients ≥65 years with ACS is associated with elevated rates of early complications, and this association is mostly driven by the increased rate of prolonged hospital stay.

This is consistent with the findings of other studies that provided evidence for a strong association between the impairment of cognitive and functional performance and worse in-hospital outcomes in patients with cardiovascular disease. In a study by Cammalleri et al., in which the prognostic role of the multidimensional prognostic index (MPI—an integrated tool for the assessment of cognitive, functional, and nutritional assessment) was examined in 241 patients ≥65 years with ACS treated with PCI, the patients with more impaired functional and cognitive performance (and higher MPI scores) had elevated rates of in-hospital complications (including longer hospital stay, a higher incidence of cardiogenic shock, new onset AF, and pneumonia) and in-hospital mortality [22]. Data from the large National Cardiovascular Data Registry—Chest Pain MI Registry that included patients with ACS ≥65 years showed that CI is associated with higher in-hospital mortality. In STEMI patients, it is 1,3-fold in the case of mild CI and 2,2-fold in the case of moderate/severe CI; in NSTEMI patients, it is 1,3-fold in the case of mild CI and 1,7-fold in the case of moderate/severe CI [23].

### The Major Adverse Cardiovascular and Cerebrovascular Events (MACCEs) at 6 Months

In our study, patients with ACS and concomitant FSxCI had an increased risk of total MACCEs at 6 months and a worse 6-month prognosis. In the aforementioned study by Cammalleri, the patients with ACS and severely impaired cognitive and functional performance had the highest 6-month mortality [21]. In a multicenter prospective cohort study by Hajduk et al. that included more than 3000 elderly patients with ACS (mean age—82 years), moderate and severe CI was associated with increased mortality at 6 months post-discharge [24].

There are several factors that may account for worse early and long-term outcomes in patients with ACS and FSxCI. These patients are older and have more comorbidities. Some studies indicate that patients with ACS and concomitant CI or FS or both are less frequently qualified for invasive treatment than patients without CI or FS—though in our study, FSxCI status was not associated with the rate and success of invasive treatment [23,24,25]. In terms of long-term prognosis, patients with cognitive and functional impairment are prone to being noncompliant with treatment and are less frequently referred for cardiac rehabilitation [23,25]. They also have an increased risk of bleeding, which challenges the optimal management of their antiplatelet therapy post-ACS. In the current European Society of Cardiology guidelines for the management of ACS, there are no specific recommendations for elderly patients that would take into account frailty syndrome and cognitive impairment [26], which is the consequence of exclusion of such patients from large randomized clinical trials. However, in patients with a high bleeding risk (and age is an important factor in bleeding scales, including PRECISE-DAPT or the Academic Research Consortium—High Bleeding Risk scales), the shortening of DAPT and the preference of clopidogrel over prasugrel or ticagrelor may be considered [26]. Antithrombotic and anticoagulant therapy in elderly post-ACS patients with FS and CI is a difficult task that requires individualization and balancing of the thrombotic and bleeding risk [27].

There is accumulating evidence that a thorough geriatric assessment of elderly patients with ACS, consisting not of only of the assessment of physical frailty but also of cognitive function, improves risk stratification and helps identify patients who require assistance in compliance with treatment, which eventually may improve their prognosis. This strategy has the potential to improve both short and long-term outcomes, make better use of resources, and tailor treatment plans to the specific needs of this vulnerable group. Further research is required to examine the role of precise geriatric assessment in ACS patients.

## 5. Conclusions

The present study demonstrates that in elderly patients with ACS, the coexistence of FS and CI (FXxCI) is frequent and is associated with worse early and 6-month prognosis. We showed an almost threefold increased risk of early complications (mainly prolonged hospital stay) and an almost threefold increased risk of MACCEs at 6 months.

*Strengths and limitations of our study*: The major strength of our study is that the presence of FS and CI was assessed during the index hospital stay and was performed via direct interview and examination with the use of validated tools, namely the Tilburg questionnaire and the MMSE. The information on FS and CI is of prognostic value. The major limitation is that it is a single-centre study performed on a relatively small sample (196 patients) and only on a Caucasian population.

## Figures and Tables

**Table 1:** Clinical characteristics of patients with ACS and with/without the coexistence of frailty syndrome and cognitive impairment (FSxCI).

	Coexistence of Frailty Syndrome and Cognitive Impairment (FSxCI)		*p*	OR [95% CI]
	Present n = 107	Absent n = 89		
**Age**, years, mean (SD)	77.9 (8.1)	70.8 (5.8)	**<0.001**	×
**Age ≥ 74 years**	76 (71.0%)	23 (25.8%)	**<0.001**	**7.04 [3.74; 13.2]**
Gender				
Female, *n* (%)	45 (42.1%)	32 (36.0%)	0.463	1.29 [0.72; 2.31]
Risk factors for CHD				
**Nicotine dependence**, *n* (%)	15 (14.0%)	26 (29.2%)	**0.013**	**0.40 [0.19; 0.80]**
**Hypertension**, *n* (%)	87 (81.3%)	55 (61.8%)	**0.004**	**2.69 [1.41; 5.14]**
Diabetes, *n* (%)	32 (29.9%)	27 (30.3%)	1.000	0.98 [0.53; 1.81]
Renal failure, *n* (%)	23 (21.5%)	19 (21.4%)	1.000	1.01 [0.51; 2.00]
COPD, *n* (%)	12 (11.2%)	6 (6.7%)	0.328	1.75 [0.63; 4.86]
Type of ACS: STEMI	53 (49.5%)	46 (51.7%)	0.776	0.92 [0.52; 1.61]
Management				
Conservative, n (%)	12 (11.2%)	6 (6.7%)	0.328	1.75 [0.63; 4.86]
Invasive, n (%)	95 (88.8%)	83 (93.3%)		
PCI, *n* (%)	85 (79.4%)	75 (84.3%)	0.460	0.72 [0.34; 1.51]
CABG, *n* (%)	10 (9.3%)	8 (9.0%)	1.000	1.04 [0.39; 2.77]
Effectiveness of PCI: optimal result (TIMI = 3)	75 (88.2%)	61 (82.8%)	0.877	1.75 [0.63; 4.86]
Time from pain onset to re-opening of artery for patients with STEMI (hours)	*n* = 53	*n* = 46	0.951	
<6 h, *n* (%)	38 (71.7%)	33 (71.7%)		1.00 (ref)
6–12 h, *n* (%)	12 (22.6%)	11 (23.9%)		0.95 [0.37; 2.43]
>12 h, *n* (%)	3 (5.7%)	2 (4.4%)		1.30 [0.21; 8.28]
Pharmacological treatment				
Beta-blocker	93 (86.9%)	83 (93.3%)	0.162	0.48 [0.18; 1.31]
ACEI/sartan	85 (79.4%)	76 (85.4%)	0.350	0.66 [0.31; 1.40]
OAC/NOAC	14 (13.1%)	7 (7.9%)	0.258	1.76 [0.68; 4.58]
	11 (10.3%)	14 (15.7%)	0.287	0.61 [0.26; 1.43]
Antiplatelet drugs ASA + P2Y12 inhibitor	106 (99.1%)	87 (97.8%)	0.592	2.44 [0.22; 27.3]
Statin	103 (96.3%)	84 (94.4%)	0.734	1.53 [0.40; 5.89]
LVEF (%), mean (SD)	44.0 (10.1)	46.5 (9.7)	0.081	×

**Table 2:** Results of univariate and multivariate logistic regression analysis.

	Univariate Logistic Regression		Mulivariate Logistic Regression		
	*b*	*p*	*beta*	*p*	*OR* [95% CI]
Age (years)	**0.135**	**<0.001**	**0.128**	**<0.001**	**1.14 [1.08; 1.19]**
Age ≥ 74 years	**1.951**	**<0.001**	-	-	-
Nicotine dependence	**−2.169**	**<0.001**	-	-	-
Hypertension	**0.977**	**0.004**	**0.783**	**0.036**	**2.18 [1.06; 4.54]**

**Table 3:** Adverse outcomes in patients with ACS and with/without coexistence of frailty syndrome and cognitive impairment (FSxCI), early and at 6 months.

	Coexistence of Frailty Syndrome and Cognitive Impairment (FSxCI)		*p*	OR [95% CI]
	Present n = 107	Absent n = 89		
Early complications, *n* (%)				
**Total early complications**	66 (61.7)	33 (37.1)	**<0.001**	**2.73 [1.53–4.88]**
Bleeding	3 (2.8%)	3 (3.4%)	1.000	0.83 [0.16–4.20]
Ventricular arrhytmia	6 (5.6%)	2 (2.2%)	0.296	2.58 [0.51–13.1
Conduction disturbances requiring cardiac stimulation	5 (4.7%)	1 (1.1%)	0.224	4.31 [0.49–37.6]
Cardiac arrest	4 (3.7%)	3 (3.4%)	1.000	1.11 [0.24–5.11]
Early stent thrombosis	2 (1.9%)	2 (2.2%)	1.000	0.83 [0.11–6.00]
Acute heart failure	10 (9.3%)	4 (4.5%)	0.267	2.19 [0.66–7.24]
Stroke	3 (2.8%)	4 (4.5%)	0.704	0.61 [0.13–2.81]
**Prolonged hospital stay (>8 days)**	56 (52.3%)	28 (31.5%)	**0.004**	**2.39 [1.33–4.30]**
In-hospital death	4 (3.7%)	2 (2.2%)	0.691	1.69 [0.30–9.45]
Major adverse cardiovascular and cerebrovascular events (MACCEs) at 6 months, *n* (%)				
**Total MACCE**	18 (16.8)	6 (6.7)	**0.047**	**2.80 [1.06–7.39]**
MI	2 (1.9%)	1 (1.1%)	1.000	1.68 [0.15–18.8]
Stroke	2 (1.9%)	0 (0.0%)	0.502	4.29 [0.20–89.5]
Recurrent revascularization	1 (0.9%)	1 (1.1%)	1.000	0.83 [0.05–13.5]
Death	14 (13.1%)	5 (5.6%)	0.093	2.53 [0.87–7.32]

## Data Availability

The data can be obtained by contacting the corresponding author.
